# Pearls of wisdom for aspiring physician-scientist residency applicants and program directors

**DOI:** 10.1172/jci.insight.158467

**Published:** 2022-03-22

**Authors:** Emily J. Gallagher, Don C. Rockey, Christopher D. Kontos, Jatin M. Vyas, Lawrence F. Brass, Patrick J. Hu, Carlos M. Isales, Olujimi A. Ajijola, W. Kimryn Rathmell, Paul R. Conlin, Robert A. Baiocchi, Barbara I. Kazmierczak, Myles H. Akabas, Christopher S. Williams

**Affiliations:** 1 Division of Endocrinology, Diabetes and Bone Disease, Department of Medicine, Tisch Cancer Institute at Mount Sinai, Icahn School of Medicine at Mount Sinai, New York, New York, USA.; 2 Division of Gastroenterology and Hepatology and Digestive Disease Research Center, Medical University of South Carolina, Charleston, South Carolina, USA.; 3 Department of Medicine, Duke University Medical Center, Durham, North Carolina, USA.; 4 Division of Infectious Disease, Department of Medicine, Massachusetts General Hospital, Boston, Massachusetts, USA; Department of Medicine, Harvard Medical School, Boston, Massachusetts, USA.; 5 Department of Medicine, Perelman School of Medicine, University of Pennsylvania, Philadelphia, Pennsylvania, USA.; 6 Departments of Medicine and Cell and Developmental Biology, Vanderbilt University Medical Center, Nashville, Tennessee, USA.; 7 Departments of Medicine, Neuroscience and Regenerative Medicine, Medical College of Georgia at Augusta University, Augusta, Georgia, USA.; 8 UCLA Cardiac Arrhythmia Center, David Geffen School of Medicine at UCLA, Los Angeles City, California, USA.; 9 Department of Medicine, Vanderbilt University Medical Center, Nashville, Tennessee, USA.; 10 VA Boston Healthcare System and Harvard Medical School, Boston, Massachusetts, USA.; 11 Division of Hematology, Department of Internal Medicine, Comprehensive Cancer Center, The Ohio State University, Columbus, Ohio, USA.; 12 Department of Microbial Pathogenesis, Department of Medicine (Infectious Diseases), Yale University School of Medicine, New Haven, Connecticut, USA.; 13 Departments of Physiology and Biophysics, Neuroscience, and Medicine, Albert Einstein College of Medicine, Bronx, New York, USA.; 14 Department of Medicine, Division of Gastroenterology, Vanderbilt University Medical Center, Nashville, Tennessee, USA; Veterans Affairs Tennessee Valley Health Care System, Nashville, Tennessee, USA; Vanderbilt Ingram Cancer Center, Nashville, Tennessee, USA.

## Abstract

Postgraduate physician-scientist training programs (PSTPs) enhance the experiences of physician-scientist trainees following medical school graduation. PSTPs usually span residency and fellowship training, but this varies widely by institution. Applicant competitiveness for these programs would be enhanced, and unnecessary trainee anxiety relieved, by a clear understanding of what factors define a successful PSTP matriculant. Such information would also be invaluable to PSTP directors and would allow benchmarking of their admissions processes with peer programs. We conducted a survey of PSTP directors across the US to understand the importance they placed on components of PSTP applications. Of 41 survey respondents, most were from internal medicine and pediatrics residency programs. Of all components in the application, two elements were considered very important by a majority of PSTP directors: (a) having one or more first-author publications and (b) the thesis advisor’s letter. Less weight was consistently placed on factors often considered more relevant for non-physician-scientist postgraduate applicants — such as US Medical Licensing Examination scores, awards, and leadership activities. The data presented here highlight important metrics for PSTP applicants and directors and suggest that indicators of scientific productivity and commitment to research outweigh traditional quantitative measures of medical school performance.

## Graphical abstract

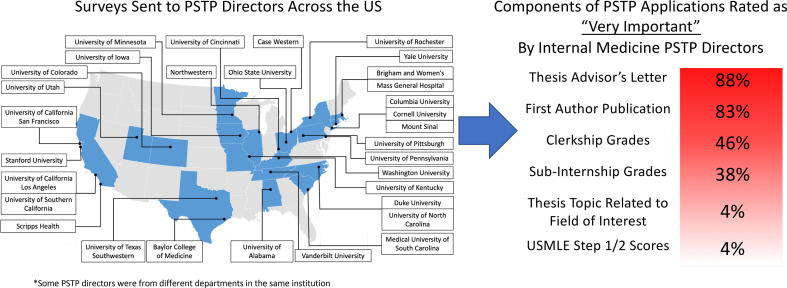

## Introduction

Physician-scientists are frequently defined as physicians whose primary activity is research. In 2012, they constituted 1.5% of the total physician workforce (1). Physician-scientists constitute a broad group with interests that encompass every aspect of medicine and a remarkable breadth of research pursuits and expertise developed through a variety of training paths; they can be found in every medical specialty/subspecialty (2). It is increasingly recognized that training obtained between medical school graduation and first faculty appointment is essential for a developing physician-scientist (3–5). Usually, this period is focused almost exclusively on achieving clinical expertise; however, nascent physician-scientists can benefit from participation in physician-scientist training programs (PSTPs) (6, 7). Although the structure of PSTPs varies widely by institution, their shared goal is to provide additional training in research and professional skills necessary for physician-scientist success (4, 7, 8). Applicant competitiveness for these programs would be enhanced by a clear understanding of what factors contribute to a successful PSTP matriculant.

A small proportion of medical school graduates enter PSTPs (9). Aspiring physician-scientists often apply to specific research tracks within clinical specialty programs, such as the American Board of Internal Medicine Research Pathway or the American Board of Pediatrics Integrated Research Pathway (3, 10). The applications of physician-scientist trainees are often reviewed by individuals focused on research, who may be distinct from the individuals who review applications of trainees applying to clinical tracks; thus, these applications are often viewed through a different lens. As a consequence, standard advice given to medical students preparing residency applications and entering “The Match” may not be appropriate for physician-scientist trainees. Physician-scientist trainees may have outstanding, successful research mentors, but these mentors may not be familiar with The Match process. Similarly, their clinical advisors may not be very familiar with the physician-scientist career pathway. Further, there may be a disconnect between the review of applicants by PSTP directors and categorical residency program directors at institutions where separate tracks exist.

To increase transparency of the PSTP selection process, we surveyed PSTP directors to gain insight into specific elements considered to be important in their decision making. In the current work, we present the results of the PSTP Applicant Evaluation Survey completed by a number of PSTP directors across the US.

## Results

### Characteristics of survey respondents and institutions.

Forty-one PSTP directors replied to the survey (of the 77 requests sent). One respondent did not identify their department affiliation or complete the questions and was excluded, leaving 40 completed surveys. Most of the 40 respondents were from internal medicine or medicine subspecialties (*n* = 24, 60%), followed by pediatrics (*n* = 9, 23%), and psychiatry (*n* = 3, 8%). There was 1 respondent from each of the following departments: anesthesiology, dermatology, pathology, and surgery. The PSTP directors represented 31 institutions across the US (some respondents were from different departments within the same institution).

As specialties vary by competitiveness, and because 2 predominant groups of respondents were from internal medicine and pediatrics, we focused our analysis on the responses of these 33 PSTP directors to provide a clearer picture of their perspectives on internal medicine and pediatrics applications. Due to the small numbers (*n* = 3), we have included the data from the psychiatry PSTP directors in Supplemental Figures 1–4 (supplemental material available online with this article; https://doi.org/10.1172/jci.insight.158467DS1).

**Figure 1:**
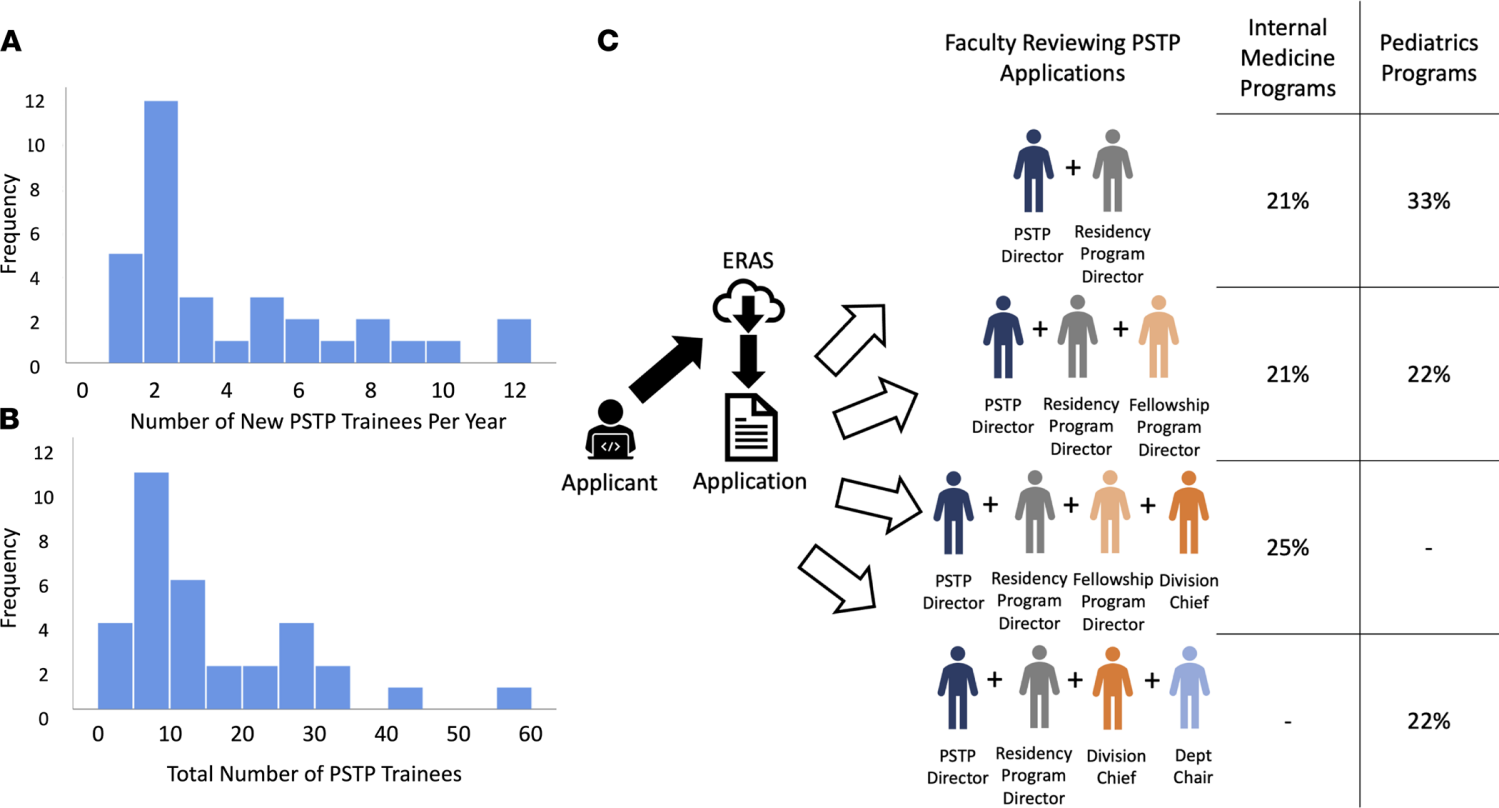
Institution affiliations, characteristics of programs, and leadership involved in evaluating postgraduate physician-scientist trainees. (**A**) Histogram depicting the number of new residents per year recruited to internal medicine and pediatrics physician-scientist training programs (PSTPs). (**B**) Histogram showing the total number of physician-scientist trainees in internal medicine and pediatrics PSTPs (bars represent groups of 5, grouped as 0–4, 5–9, 10–14, etc.). (**C**) Schematic representing faculty leadership who most commonly review internal medicine and pediatrics applications submitted to PSTPs through the Electronic Residency Application Service (ERAS).

For the internal medicine and pediatrics PSTPs, the median number of new PSTP residents per year was 2 (range 1–12 residents/year, Figure 1A), with similar numbers for internal medicine (median 2.5, range 1–12 residents/year) and pediatrics (median 2, range 1–9 residents/year). The median total number of postgraduate physician-scientist trainees in these programs was 12 (range 1–57 residents/year, Figure 1B), with a median of 12 for the internal medicine (range 1–40 residents/year) and 8 for the pediatrics (range 1–57 residents/year) programs.

### Structure of the application selection process.

Fifteen internal medicine (63%) and 6 pediatrics (67%) PSTP directors reported that their programs had a separate Electronic Residency Application Service (ERAS) match number for their research and clinical residency programs. Thirteen internal medicine (54%) and all 9 (100%) pediatrics directors reported that PSTP applications were identified and separated from other applications during the selection process. The majority of PSTP directors (10 of 13 internal medicine, and 8 of 9 pediatrics) who reported separation of PSTP applications from the larger pool indicated that these applications underwent additional review and/or were specifically reviewed by the PSTP director.

For most programs, physician-scientist trainee applications were adjudicated by a combined group of departmental leaders (Figure 1C). The most common combination in internal medicine was the PSTP director, residency program director, fellowship program director, and division chief (*n* = 6, 25%). In pediatrics, this combination was most commonly the PSTP director and residency program director (*n* = 3, 33%). Five internal medicine (21%) and 3 pediatrics (33%) PSTP directors indicated that someone else was involved in the selection of applicants, which included a committee and/or other faculty interviewers.

### Applicant as a scientist — productivity and research funding.

**Figure 2:**
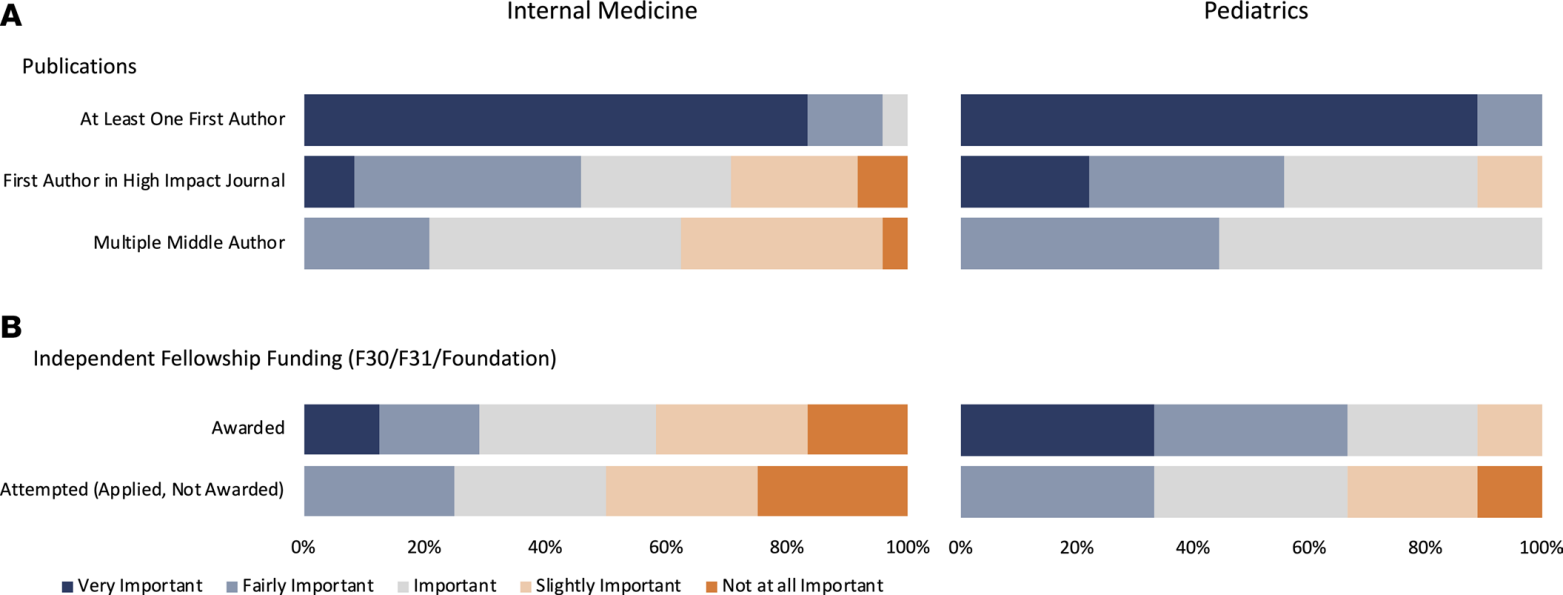
Importance of academic performance — publications and funding. Responses of internal medicine (*n* = 24) and pediatrics (*n* = 9) physician-scientist training program (PSTP) directors to survey questions are shown as stacked bar graphs. PSTP directors were asked to rate each item as very important (dark blue), fairly important (light blue), important (gray), slightly important (light orange), or not at all important (orange), as shown. (**A**) Importance placed on publications. (**B**) Importance of obtaining or applying for funding.

Having at least 1 first-author publication was reported as very or fairly important by 96% of internal medicine and 100% of pediatrics PSTP directors (Figure 2A). Approximately one-half of the PSTP directors in both internal medicine and pediatrics considered a first-author publication in a high impact journal to be very important or fairly important, but 29% of internal medicine directors considered it slightly important or not at all important and 11% of the pediatrics PSTP directors considered it slightly important. Five internal medicine (21%) and 4 pediatrics (44%) PSTP directors ranked multiple middle-author publications as fairly important, but none considered it very important.

Opinions of PSTP directors regarding the importance of receiving previous research funding (e.g., F30, F31, or foundation funding) appeared to be divided in internal medicine, with 12.5% (*n* = 3) considering it very important and 17% (*n* = 4) considering it not at all important (Figure 2B). In contrast, 67% (*n* = 6) of pediatrics PSTP directors considered previous research funding to be very important or fairly important, and none considered it not at all important. None of the PSTP directors considered attempting to obtain research funding (applied, but not awarded) to be very important, while one-quarter of internal medicine PSTP directors and 11% of pediatrics PSTP directors rated it not at all important (Figure 2B).

### Applicant as a clinician — medical knowledge and clinical skills.

**Figure 3:**
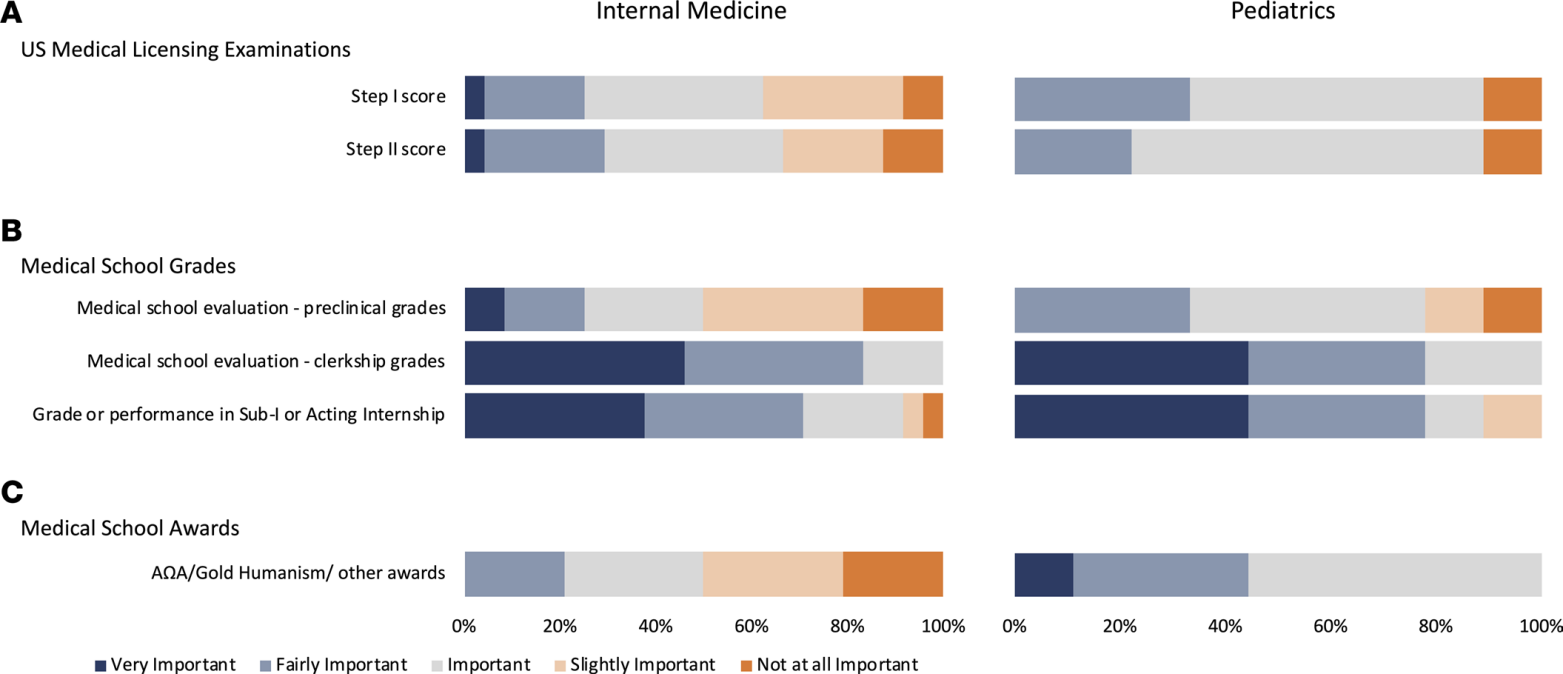
Board scores and clinical performance. Responses of internal medicine (*n* = 24) and pediatrics (*n* = 9) physician-scientist training program (PSTP) directors to survey questions are shown as stacked bar graphs. PSTP directors were asked to rate each item as very important (dark blue), fairly important (light blue), important(gray), slightly important (light orange), or not at all important (orange), as shown. (**A**) Importance placed on US Medical Licensing Examination scores. (**B**) Importance of medical school grades. (**C**) Importance of other medical school awards.

Only 1 internal medicine PSTP director and no pediatrics PSTP directors considered US Medical Licensing Examination (USMLE) Step 1 scores as very important (Figure 3A). On this issue, internal medicine PSTP directors were roughly evenly divided among fairly important, important, and slightly important. However, only 2 internal medicine PSTP directors (8%) felt that Step 1 scores were not at all important. More than one-half of pediatrics PSTP directors rated USMLE Step 1 scores as important, with one (11%) considering them not at all important.

The PSTP directors’ opinions were similarly distributed on the importance of USMLE Step 2 scores (Figure 3A). One internal medicine PSTP director (4%) and no pediatrics PSTP directors considered them very important, while 3 internal medicine (13%) and 1 pediatrics PSTP director (11%) considered them not at all important. There was a wide distribution in opinions regarding the importance of preclinical grades. Two internal medicine PSTP directors (8%) considered them very important, and 4 (17%) considered them not at all important. No pediatrics PSTP director considered them very important, and 1 (11%) considered them not at all important (Figure 3B)

The attitudes toward the importance of clinical grades contrasted with those toward the preclinical grades (Figure 3B). Clerkship grades were considered very important or fairly important by 83% of internal medicine PSTP directors (*n* = 20), and subinternship grades were considered very important or fairly important by 71% (*n* = 17). Similarly, clerkship and subinternship grades were rated as very important or fairly important by 78% of pediatrics PSTP directors. No internal medicine PSTP directors considered Alpha Omega Alpha (AΩA) membership and/or humanism in medicine awards to be very important, and 21% (*n* = 5) reported they were not at all important (Figure 3C). Five (55%) pediatrics PSTP directors rated these as important, and 44% (*n* = 4) considered them to be very important or fairly important.

### Letters of support.

**Figure 4:**
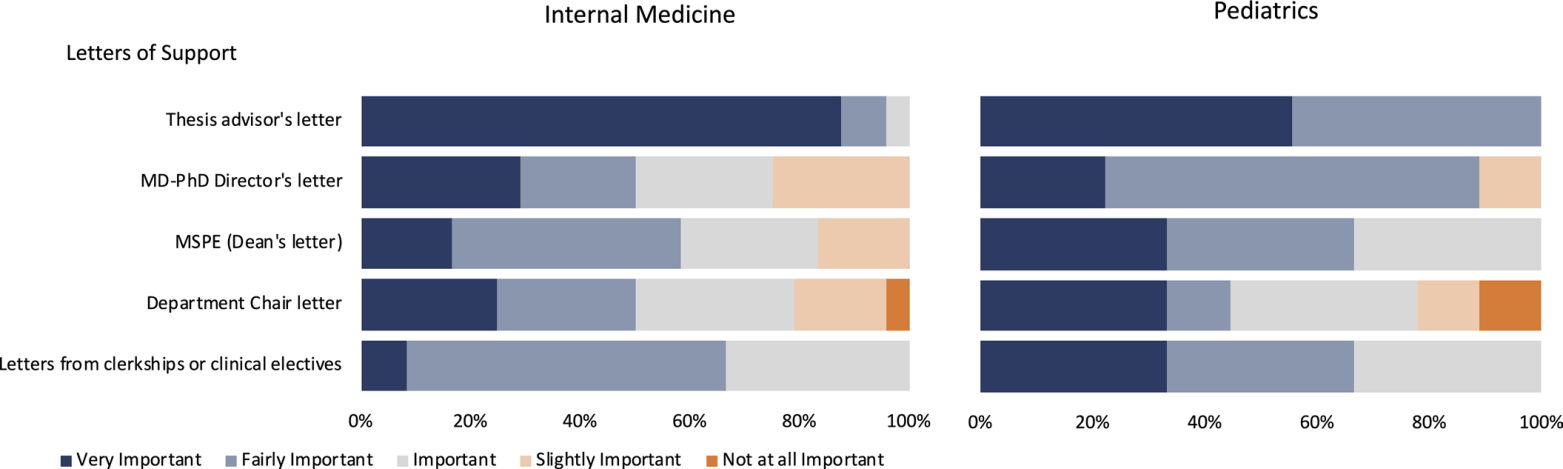
Letters of support and other factors considered important. Responses of internal medicine (*n* = 24) and pediatrics (*n* = 9) physician-scientist training program (PSTP) directors to survey questions are shown as stacked bar graphs. PSTP directors were asked to rate each letter of support as very important (dark blue), fairly important (light blue), important (gray), slightly important (light orange), or not at all important (orange), as shown. MSPE, medical student performance evaluation.

The letters of support from the MD-PhD Director, dean (medical student performance evaluation [MSPE]), thesis advisor, and clerkship and clinical elective supervisors were viewed as very important or fairly important by the majority of internal medicine and pediatrics PSTP directors, with no one considering them not at all important (Figure 4). When available, the thesis advisor’s letter was considered the most important, with 88% of internal medicine and 56% of pediatrics PSTP directors ranking it as very important. The department chair’s letter was generally not rated as high in importance as the other letters by either internal medicine or pediatrics PSTP directors.

### The person, the fit, and the plan.

**Figure 5:**
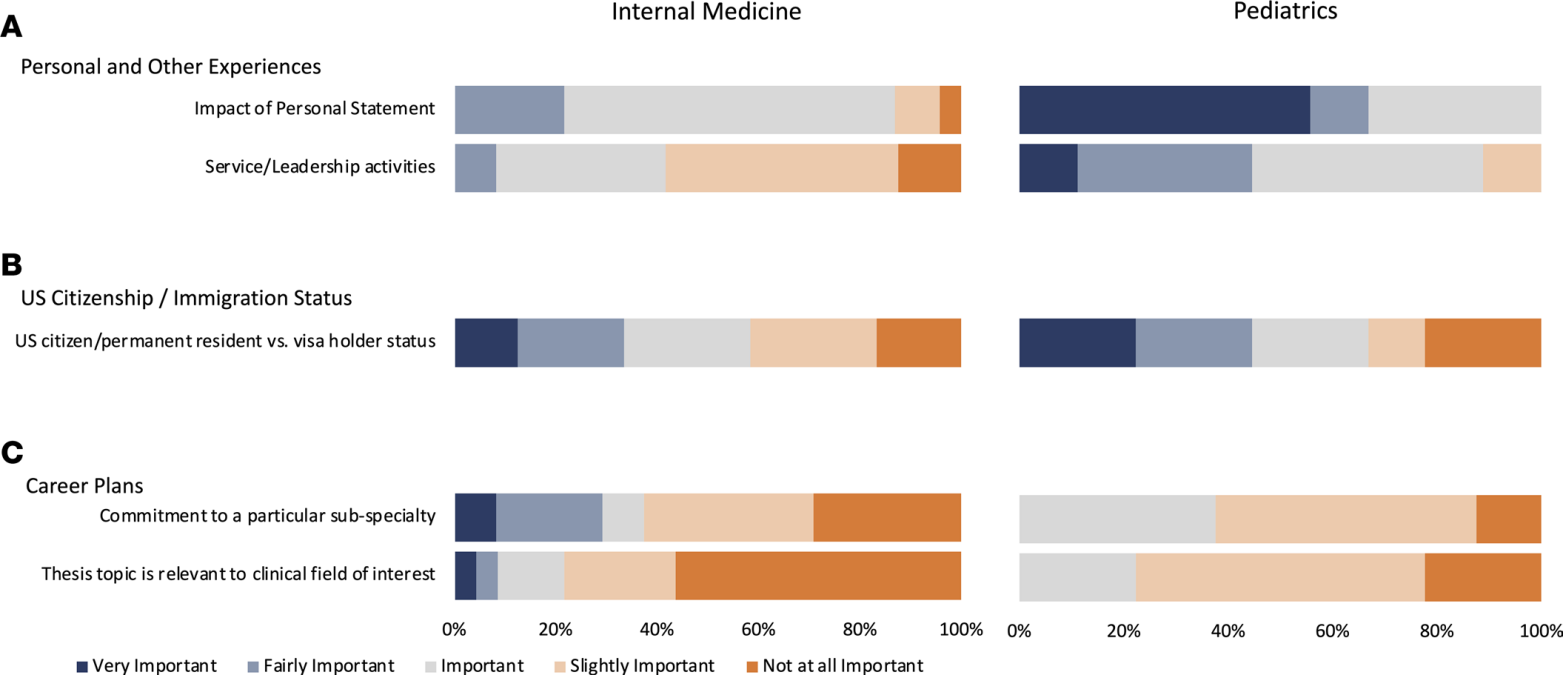
Personal statement, leadership, citizenship, and future plans. Responses of internal medicine and pediatrics physician-scientist training program (PSTP) directors on the subjects of personal and other experiences (**A**); US citizenship/immigration status (**B**); and career plans (**C**), are presented as stacked bar graphs. PSTP directors were asked to rate each item as very important (dark blue), fairly important (light blue), important (gray), slightly important (light orange), or not at all important (orange), as shown. Note that *n* = 23 internal medicine PSTP directors responded to the questions on “Thesis topic is relevant to the clinical field of interest” and “Impact of personal statement.” There were *n* = 24 internal medicine respondents for the other questions and *n* = 9 pediatrics PSTP director respondents.

The applicant’s personal statement was considered important by 65% of internal medicine PSTP directors, with none considering it very important and 4% considering it not at all important (Figure 5A). Less importance appeared to be placed on service/leadership activities by internal medicine PSTP directors, with none considering them very important and a majority considering them slightly important or not at all important (Figure 5A). In contrast, 56% of pediatrics PSTP directors considered the applicant’s personal statement to be very important, and more than one-third rated service/leadership activities as very important or fairly important.

The PSTP directors in both internal medicine and pediatrics had divided attitudes toward the importance of citizenship or permanent resident status, with similar proportions considering it very important and not at all important (Figure 5B). Of the 5 programs (3 internal medicine, 2 pediatrics) that considered US citizenship or permanent resident status to be very important, none reported accepting international medical graduates.

Only 8% (*n* = 2) of internal medicine PSTP directors considered commitment to a particular subspecialty to be very important, while no pediatrics PSTP directors considered it very important or fairly important (Figure 5C). Over 50% of internal medicine PSTP directors and 22% of pediatrics PSTP directors thought that it was not at all important for the applicant’s thesis topic to be relevant to their clinical interest (Figure 5C). One internal medicine PSTP director (4%) thought that it was very important and 1 (4%) thought it was fairly important that the trainee’s thesis topic was relevant to their clinical field of interest, while none of the pediatrics PSTP directors thought it was very important or fairly important.

## Discussion

With this survey, we have shed light on important variables that PSTP directors consider in selecting candidates for their programs. We have divided the Discussion into sections to emphasize critical insights for the two major stakeholders (i.e., applicants and PSTPs) in this process. We have also included a section in the Discussion targeted toward the PSTP directors who evaluate applications as well as a section with some strengths, limitations, and potential future research needed in this field.

### For physician-scientist applicants.

The first thing to note is the commitment that institutions have to selecting the candidates for physician-scientist training. Many of the senior leadership teams involved in reviewing the PSTP applications are likely made up of physician-scientists, and we posit that their involvement in the review process indicates their commitment to training the next generation of physician-scientists. Leadership faculty at training institutions have many years of combined experience in training physician-scientists. Based on these experiences, we assume that they strive to identify applicants who are most committed to careers as physician-scientists and who are in their view the best equipped to be successful.

Applicants having at least 1 first-author peer-reviewed paper was viewed as very important by almost all of the PSTP directors in internal medicine and pediatrics. In contrast, there was less agreement on whether that paper had to be published in a high-impact journal. Furthermore, while having multiple coauthored papers was generally viewed as supportive, none of the PSTP directors thought it was very important. It is also likely that PSTP applications are viewed from a different perspective than those of non-PSTP applicants. For example, for an MD-PhD graduate, having published a first-author manuscript during MD-PhD training may be viewed to be extremely important; however, for an MD-only applicant this would probably not be considered as important. It is important to recognize some of the values and expectations that may be behind these views. First-author publication may be viewed as a surrogate for the ability of an applicant to see a project through to completion. First-author publication also marks an individual as someone who has experienced the process of discovery and creating new knowledge. These qualities are difficult to assess in other ways, which may be a reason why much emphasis is placed on productivity in graduate training.

The second major point is that clinical clerkship and sub/acting internship grades are considered very or fairly important for the evaluation of these applicants, whereas preclinical grades and USMLE Step 1 and 2 scores are less important. Going forward, USMLE Step 1 scores will be irrelevant as the examination became pass/fail in January 2022. However, only a small minority of PSTP directors considered these scores very important, so the effect of this change on physician-scientist trainee applications to internal medicine and pediatrics PSTPs will likely be minimal. Training programs are held to various standards to maintain their accreditation and to ensure that the physicians who emerge from their programs meet basic clinical competencies. Grades in clinical clerkships may be viewed as better indicators of clinical competency, as compared with standardized tests or preclinical grades.

The third key point is that almost 80% of both internal medicine and pediatrics PSTP directors thought it was not at all important or slightly important that trainees pursue a clinical field that was related to their research topic. This suggests that a majority of PSTP leadership believes that the core scientific principles learned during research training are adaptable to any medical/scientific field. This point is particularly important, as this question is often raised by trainees and appears to influence their choice of specialty.

It should be emphasized that applications are a marketing document, i.e., a means for an applicant to accentuate key elements of their accomplishments to program leadership and to convey a sense of commitment to and potential for a successful physician-scientist career. It is essential to illustrate how one’s previous training will lay the foundation for future success. For example, many students are often encouraged to have a broad scope of activity during medical school training; however, most physician-scientists have been successful because they have been highly focused, a trait that can be fostered during training. As physician-scientists are meeting a different need for programs, the range of portfolio models may be different for someone pursuing a career as a physician-scientist than for a standard MD/DO applicant. The key for all applicants is to differentiate themselves in a way that fits well with the program to which they are applying. It is important to provide an honest portrayal of strengths and skills to show a program what assets a candidate will bring to the program, particularly for a program that is seeking to develop physician-scientists.

It is also imperative to identify specific criteria that may influence the review of a prospective trainee’s application. For example, some institutions rely on federal grants to support the training of physician-scientists during their postgraduate years; therefore, these institutions may accept only trainees who are US citizens or permanent residents who can be supported on these grants. There were differences in the responses between internal medicine and pediatrics PSTP directors regarding the importance of AΩA/Gold Humanism awards, leadership activities, and the personal statement. Therefore, it is important to consider these differences when applying to different residency programs. Although a large number of applicants to PSTPs hold dual-doctoral degrees (MD-PhD), in the comments section of the survey, most programs reported considering both medical-degree only as well as dual-doctoral degree (MD-PhD) applicants. In these instances, the research experience and portfolio of research products can carry as much weight as a PhD.

### For physician-scientist advisors.

In the comments section of the survey, the PSTP directors cited a commitment to science as being a critical aspect of the applicant’s evaluation. Thus, it is important to note that significant emphasis was placed on the letter of recommendation from the thesis advisor; additionally, it was considered critical for the trainee to have a first-author publication. Resilience is an important quality for successful physician-scientists, but sometimes this is apparent only through the observations of their thesis advisor. Frequently, the thesis advisor’s letter is the only place in the application where these qualities can be discerned; we postulate that composing a letter with specific anecdotes and an honest evaluation of the PSTP applicant’s abilities will help those adjudicating applications. PSTP directors were less concerned about whether the area of the trainee’s thesis is relevant to their subsequent clinical discipline. Rather, it appeared that directors placed more emphasis on receiving high-quality research training that can be translated to any specialty. Of note, PSTP directors also emphasized the importance of a first-author publication per se and were less concerned about having a first-author publication in a high-impact journal. Posting a manuscript on a preprint server, such as BioRχiv or MedRχiv, that can be cited by the applicant could help fulfill this important benchmark (11) and provide PSTP directors with tangible evidence of research productivity in the absence of a published paper. The challenge of when and where to publish is faced by every researcher. Many thesis advisors, for the sake of their own career trajectory, may strongly encourage trainees to seek publication in higher-tiered journals. From the perspective of a trainee’s career development, this approach may not serve them well, and the adage “perfect is the enemy of good” appears to hold true.

### For PSTP directors.

In this survey of PSTP directors, there were areas with a general consensus regarding levels of importance (e.g., the thesis advisor’s letter and any first-author publication) and areas where opinions differed within specialties (e.g., the importance of receiving funding and commitment to a particular subspecialty) and between specialties (e.g., the importance of the personal statement and leadership activities). These diverging opinions may represent the experiences of PSTP directors who trained previous physician-scientists; there is potential that additional items could enhance an application but would not substitute for the attributes considered to be very important. At the present time, PSTPs do not typically publicize the attributes that they consider important in their application selection process. Going forward, transparency regarding the components of applications that specific programs consider most important, particularly among the items where PSTP responses were mixed, could remove some of the guesswork for applicants and their advisors. While the questions presented in this survey were based on the preformatted ERAS application, which is currently the primary tool used to review PSTP applicants, it remains to be determined whether this form truly presents the criteria that capture the potential success of future physician-scientists.

### Strengths and limitations.

The survey had an overall response rate of 52%, which is generally considered excellent, especially in surveys of physicians. The survey had a number of strengths. By focusing on the responses from internal medicine and pediatrics PSTPs, which represented the largest 2 groups of respondents, we aimed to create a clearer picture of what is considered important by PSTP directors in these fields. This could benefit future physician-scientist applicants to internal medicine and pediatrics PSTPs. We have also included the responses from the small number of psychiatry PSTP directors (Supplemental Figures 1–4). We identified some differences between the internal medicine and pediatrics PSTP directors. Going forward, profiling the similarities and differences in attitudes of PSTP directors across different specialties would be useful for all graduating medical students and their advisors, because only about 25% of MD-PhD program graduates train in internal medicine and 13% in pediatrics (12). A limitation of the survey was that it did not include outcomes for trainees from PSTP programs, so whether the importance of the selection criteria reported by these PSTP directors is related to their trainees’ retention or long-term success as physician-scientists is unknown. The survey focused on elements included within the ERAS application at the time the survey was conducted. Of note, there were some areas where opinions were divided regarding importance, although through the medium of the survey, we were unable to delve into the reasons behind these differing opinions. Finally, the importance of diversifying the pool of physician-scientists was not addressed in this survey, although it was highlighted as important by some PSTP directors in the comments section.

### Conclusions.

Overall, the results of our survey of PSTP directors suggest that physician-scientist applicants to internal medicine and pediatrics programs are likely best served by focusing on their research training, obtaining strong letters of support (particularly from their thesis advisor) that provide a clear appraisal of the applicants’ capabilities, and publishing their research in a first-author publication. Performance in the clinical training phase is also important, although preclinical grades do not appear to be highly important. These survey results provide a critical foundation for physician-scientist trainees, their advisors, and PSTP directors and should help create a clearer application pathway for success for emerging physician-scientists.

## Methods

The survey was distributed via email to Directors of Research in residency programs and PSTPs between November 18, 2019, and January 30, 2020. Programs were identified by combining Alliance of Academic Internal Medicine Registries of PSTP directors and a direct examination of internal medicine residency websites for PSTP contact information. The survey questions are shown in Table 1.

### Statistics.

Most responses used a 5-point Likert scale, with possible responses being very important, fairly important, important, slightly important, and not at all important. Seventy-seven surveys were distributed, and there were 41 respondents (53.2%). Results are presented as numbers and percentages. Graphs were generated using SPSS V26 (IBM) or Microsoft Excel for Mac V16.53.

### Study approval.

IRB approval was obtained from Vanderbilt University for the project, entitled PSTP Applicant Evaluation Survey (IRB protocol 191912).

## Author contributions

CDK, JMV, LFB, PJH, CMI, OAA, MHA and CSW designed the study. EJG analyzed the data. DCR, EJG, and CSW initially drafted the manuscript. All authors wrote and edited the manuscript.

## Supplementary Material

Supplemental data

## Figures and Tables

**Table 1:**
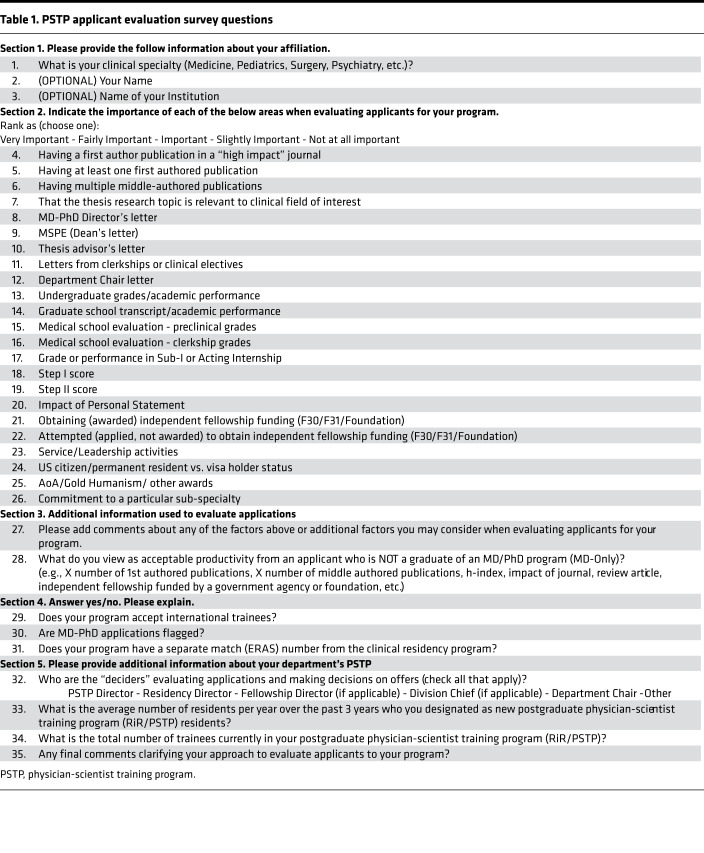
PSTP applicant evaluation survey questions

## Version 1 03/22/2022

Electronic publication
