# Supplementary material for: Pearls of wisdom for aspiring physician-scientist residency applicants and program directors
Source: JCI Insight. 2022 Mar 22;7(6):e158467. doi: 10.1172/jci.insight.158467 (PMC8986063; doi:10.1172/jci.insight.158467)
Supplement: Supplemental data [file jciinsight-7-158467-s256.pdf]

Supplemental material.

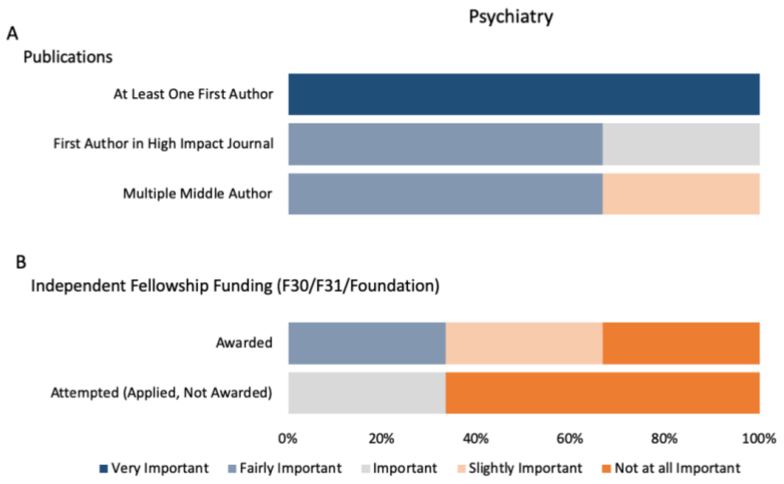

Supplemental Figure 1. Importance of academic performance - publications and funding from psychiatry PSTP directors. Responses of psychiatry PSTP directors (n=3) to survey questions are shown as stacked bar graphs. PSTP directors were asked to rate each item as very important, fairly important, important, slightly important or not at all important, as shown. A. Importance placed on publications. B. Importance of obtaining or applying for funding.

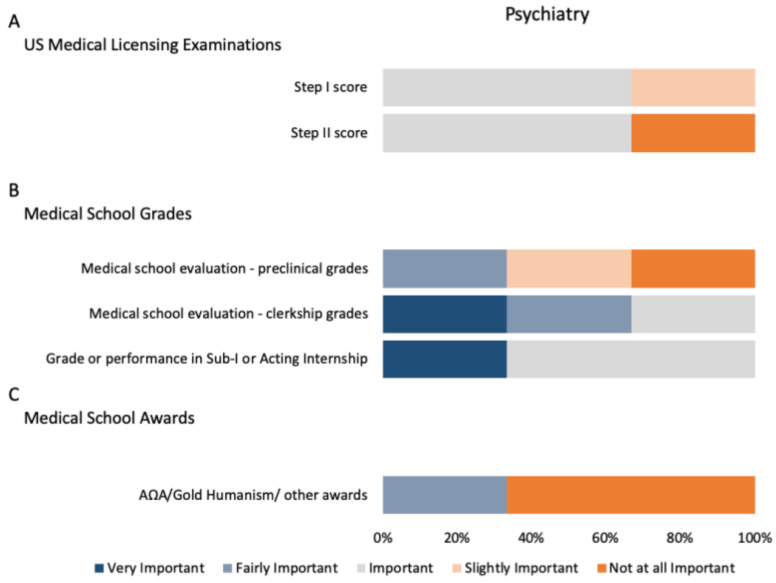

Supplemental Figure 2. Board scores and clinical performance from psychiatry PSTP directors. Responses of psychiatry PSTP directors (n=3) to survey questions are shown as stacked bar graphs. PSTP directors were asked to rate each item as very important, fairly important, important, slightly important or not at all important, as shown. A. Importance placed on USMLE scores. B. Importance of medical school grades. C. Importance of other medical school awards.

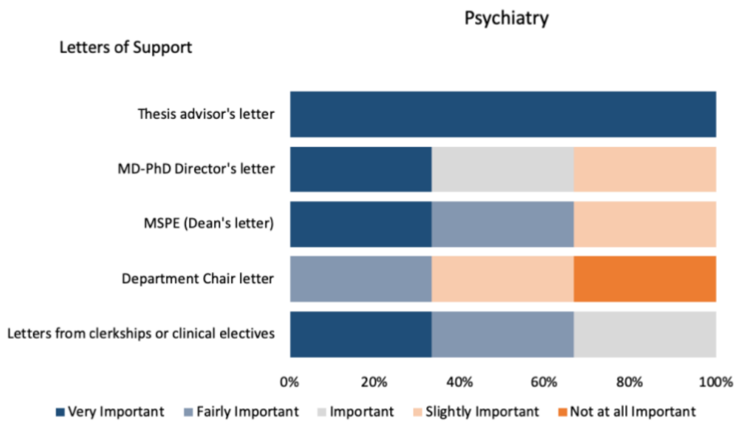

Supplemental Figure 3. Letters of support and other factors considered important by psychiatry PSTP directors. Responses of psychiatry PSTP directors (n=3) to survey questions are shown as stacked bar graphs. PSTP directors were asked to rate each letter of support as very important, fairly important, important, slightly important or not at all important, as shown.

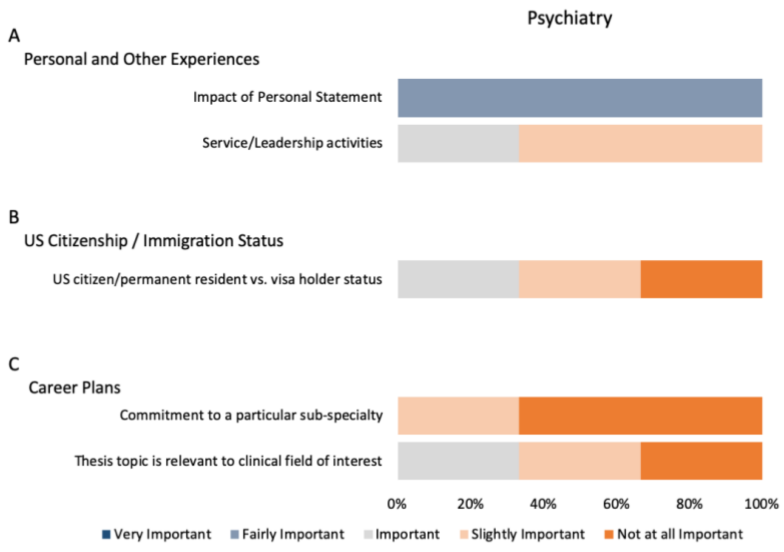

Supplemental Figure 4. Personal statement, leadership, citizenship, and future plans. Responses of psychiatry PSTP directors (n=3) are presented as stacked bar charts. PSTP directors were asked to rate each item as very important, fairly important, important, slightly important or not at all important, as shown.
